# A Comprehensive Review of Phototherapy in Atopic Dermatitis: Mechanisms, Modalities, and Clinical Efficacy

**DOI:** 10.7759/cureus.56890

**Published:** 2024-03-25

**Authors:** Amr Molla

**Affiliations:** 1 Department of Medicine, Taibah University, Madinah, SAU

**Keywords:** puva, uva, broadband uvb, narrowband uvb, phototherapy, pathogenesis, atopic dermatitis

## Abstract

This literature review explores atopic dermatitis and its management, with a focus on phototherapy as a treatment modality.

The primary objectives are to elucidate the pathophysiological mechanisms, clinical manifestations, diagnostic criteria, and epidemiology of atopic dermatitis. Additionally, it seeks to explain phototherapy mechanisms, different modalities, and other therapeutic approaches.

In this review, we comprehensively examine atopic dermatitis by synthesizing findings from diverse sources over the past 20 years. We investigate the epidemiology, pathophysiology, clinical manifestations, diagnostic criteria, and role of phototherapy in treatment. We conduct thematic analysis, compare phototherapy modalities, consider contextual factors, and integrate patient perspectives while upholding ethical considerations. Limitations include potential publication bias, language barriers, temporal constraints, subjectivity, and limited generalizability.

Atopic dermatitis has a complex pathogenesis and can be managed with diverse modalities. Phototherapy emerges as an effective and safe treatment, particularly when other therapies prove ineffective.

## Introduction and background

Atopic dermatitis (AD) is a common, pruritic, and chronic inflammatory skin condition that typically commences in childhood and frequently persists into adulthood. It exhibits a pattern of relapses and remissions and exerts a significant impact on patients' quality of life [1,2]. AD appears to have a multifactorial etiology, involving genetic, environmental, and immunological factors. The prevalence of AD varies according to the demographic characteristics of the population studied. Globally, AD is reported to affect approximately 3-30% of children and 1-10% of adults. In developed nations, the prevalence is estimated at 10-20% in children and 1-3% in adults, while in developing countries, the rates range from 1% to 10% in children and less than 1% in adults [3].

The primary goal of AD treatment is to achieve disease control, minimize skin rash and pruritus, and enhance patients' quality of life through personalized strategies [1]. Various factors, including age, behavioral and socioeconomic considerations, patient adherence, and psychological well-being, need to be taken into account [4]. While many patients find relief through initial measures such as gentle cleansing, emollients, and allergen avoidance, some individuals may require systemic therapies. Phototherapy is employed during acute exacerbations and in cases where chronic AD remains uncontrolled despite topical treatments [5].

Phototherapy is considered a second-line treatment option when conventional and topical therapies prove ineffective for both chronic and acute AD in both children and adults [3]. In severe, widespread AD cases, it may even be utilized as a first-line treatment modality. Phototherapy involves the therapeutic use of non-ionizing radiation, particularly within the ultraviolet (UV) spectrum, to alleviate symptoms [6]. Despite the advent of potent biological treatments, UV phototherapy continues to be an established, cost-effective, and efficacious therapeutic approach for various inflammatory skin conditions characterized by T-cell-rich infiltrates, such as AD [1,2].

The objective of this literature review is to provide physicians, particularly dermatologists, with an overview of the diverse therapeutic approaches used in the routine management of various cutaneous diseases. It's important to note that, as of now, there have been no suitably sizable randomized controlled studies and no single light modality has been conclusively proven to be superior to others [7]. Therefore, it is crucial to emphasize the efficacy and safety of phototherapy in the treatment of AD to raise awareness and educate the population.

## Review

Methodology

Objective

Our aims in this study are to review AD epidemiology globally, understand AD's pathophysiology and phototherapy's role, examine AD's clinical signs and its diagnostic criteria, analyze phototherapy's action mechanisms and compare efficacy and safety across modalities, evaluate phototherapy's current evidence for AD treatment, considering patient perspectives, and identify research gaps, focusing on long-term effects, treatment optimization, and phototherapy integration with other treatments.

Search Strategy

We conducted a comprehensive literature review, analyzing 37 articles from databases like PubMed, Scopus, and Web of Science, covering the past 20 years. We focused on study design, outcomes, and relevant findings to understand AD and phototherapy's role, organizing the synthesis around emerged key themes like pathophysiology, clinical signs, diagnostics, and phototherapy modalities.

Synthesis of Findings

We aimed to integrate study findings to draw conclusions on AD and phototherapy, exploring themes such as pathophysiology, clinical manifestations, and diagnostics. Our thematic analysis identified patterns and discrepancies, with a comparative evaluation of phototherapy modalities for efficacy, safety, and patient demographics. We contextualized findings by geographic and demographic variations and included patient perspectives for a holistic view of AD's impact and treatment.

Ethical Considerations

We emphasized academic integrity through proper citation and acknowledgment, ensuring confidentiality and data protection. We aimed for an objective presentation free from plagiarism, bias, or external influence.

Limitations of the Methodology

We acknowledge limitations such as publication bias, language barriers excluding non-English studies, temporal limitations missing recent advances, and the inherent subjectivity in data interpretation. The generalizability of findings may be restricted by the geographic scope and demographics of the reviewed studies.

Epidemiology of AD

Epidemiological investigations have unveiled the prevalence of AD, commonly referred to as eczema, within the region. For adult patients, the diagnosis of AD was established based on the UK Working Party criteria and self-report of a physician's AD diagnosis. These studies identified a noteworthy prevalence rate of AD, reaching 15.3%. It is noteworthy that significantly higher rates were observed among females and individuals aged 45-54 years. Among adults with AD, rhinitis emerged as the most prevalent comorbidity, with a striking prevalence rate of 91.3% [1,3,8].

In the pediatric population, the 12-month prevalence of doctor-diagnosed eczema was determined based on the criteria of the International Study of Asthma and Allergies in Childhood (ISAAC) in conjunction with self-reported information. The prevalence rates exhibited variations among different age groups. Children aged six months to less than six years had a lower prevalence of 11.3%, whereas those aged six to less than 12 years showed a prevalence rate of 18.3%, and those aged 12 to less than 18 years had the highest rate at 29.4% [9].

The burden of this chronic inflammatory skin condition is substantial, with the total economic cost of AD constituting up to 0.059% of the country's gross domestic product (GDP), ranking among the highest worldwide [10]. Generally, AD exhibits a higher prevalence among the urban population and is less prevalent in rural areas. Moreover, it is more commonly observed among individuals with higher socioeconomic status and those with elevated levels of family education [8].

Understanding the epidemiology of AD is of paramount importance, as it paves the way for targeted interventions and the enhancement of condition management strategies.

Pathogenesis of AD

The disease is linked to high serum immunoglobulin (IgE) levels and a history of type I allergies, allergic rhinitis, and asthma, reflecting its complex, multifactorial pathogenesis. This includes skin barrier dysfunction, environmental influences, genetic predisposition, and immune system abnormalities [1].

To date, 46 genes associated with AD have been identified, influencing keratinocyte differentiation and immune responses. Notably, mutations in the filaggrin (FLG) genes are common, affecting skin protein filaggrin expression. Other significant genes include those coding for claudins, loricrin, involucrin, SPINK5, and TMEM79/MATT, which impact skin barrier functions [11]. Mutations affecting the innate immune system (e.g., NOD1, NOD2, TLR2, CD14, DEFB1) and adaptive immunity (e.g., IL-4, IL-4RA, IL-13, TSLP, IL-31, CCR5) have been linked to AD, highlighting the role of genetic and environmental factors in its development [12]. In AD, both affected and unaffected skin shows reduced levels of key structural proteins and lipids critical for barrier integrity and hydration. The stratum corneum's structure, comprised of proteins, like loricrin and involucrin, and a lipid matrix, is compromised, as is tight junction functionality, particularly claudins [11].

FLG gene mutations disrupt skin barrier formation by affecting filaggrin production, increasing vulnerability to allergens and transepidermal water loss [13]. Furthermore, cytokines from Th2 cells, such as IL-4 and IL-13, weaken the skin barrier and antimicrobial defenses, with Th22 and IFN gamma involvement in barrier disruption and inflammation. AD's immune response transitions from a Th2 to a Th1 profile in chronic stages, illustrating the dynamic nature of its pathophysiology [14].

Clinical manifestations, complications, and diagnostic criteria of AD

AD manifests in three distinct phases, acute, subacute, and chronic, with symptoms ranging from vesicular eruptions to lichenification due to persistent scratching. It typically affects areas like the flexures, neck, eyelids, and extremities. Diagnosis is challenging, relying on clinical signs, lesion distribution, and historical features rather than specific tests. The 1980 Hanifin and Rajka criteria, requiring a combination of major and minor criteria, serve as a standard diagnostic tool but are often cumbersome for practical use due to their complexity and the inclusion of vague minor criteria [1,3,15].

Various formal sets of criteria have been established as classification tools, with the 1980 Hanifin and Rajka criteria being one of the earliest and most widely used (Table 1) [15]. These criteria demand the fulfillment of three of four major criteria and three of 23 minor criteria. While comprehensive and commonly used in clinical trials, the large number of criteria proves inconvenient for clinical practice. Some minor criteria are vague or non-specific, such as pityriasis alba, while others, like nipple eczema and upper lip cheilitis, are quite specific to AD but uncommon. To address these limitations, several international groups have proposed modifications. Notably, the UK Working Party streamlined the Hanifin and Rajka criteria (Table 1) into a core set suitable for epidemiological population-based studies and use by non-dermatologists. These criteria include one mandatory and five major criteria and do not necessitate laboratory testing [16].

**Table 1:** The Hanifin and Rajka criteria for the diagnosis of atopic dermatitis Reference: [15]

Serial no.	Major criteria	Minor criteria
1	Pruritus	Xerosis
2	Typical morphology and distribution: flexural lichenification in adults; facial and extensor eruptions in infants and children	Ichthyosis/palmar hyperlinearity, keratosis pilaris
3	Chronic or chronically relapsing dermatitis	Immediate (type I) skin test reaction
4	Personal or family history of atopy (asthma, allergic rhinitis, atopic dermatitis)	Elevated serum IgE
		Early age of onset
		Tendency toward cutaneous infections (especially *Staphylococcus aureus* and herpes simplex), impaired cell-mediated immunity
		Tendency toward non-specific hand or foot dermatitis
		Nipple eczema
		Cheilitis
		Recurrent conjunctivitis
		Dennie-Morgan infraorbital fold
		Keratoconus
		Anterior subcapsular cataracts
		Orbital darkening
		Facial pallor, facial erythema
		Pityriasis alba
		Anterior neck folds
		Itch when sweating
		Intolerance to wool and lipid solvents
		Perifollicular accentuation
		Food intolerance
		Course influenced by environmental and emotional factors
		White dermographism, delayed blanch

The assessment of AD severity involves evaluating both clinical signs and subjective symptoms. The Scoring Atopic Dermatitis Index is the most widely employed and validated clinical tool for grading AD severity based on the affected body area and the intensity of lesion characteristics. Other validated tools include the Eczema Area and Severity Index, the Investigator Global Assessment Scale, and the Six Area, Six Sign Atopic Dermatitis (SASSAD) Severity Score [17].

Furthermore, AD predisposes patients to secondary bacterial infections, predominantly from *Staphylococcus* and *Streptococcus*, manifesting as pustules or crusts. Additionally, individuals with AD are at risk of herpes simplex virus (HSV) infections, referred to as Kaposi varicelliform eruption or eczema herpetic. Eczematous skin provides a platform for localized herpes outbreaks, resulting in a painful papulovesicular rash. Picking or scratching of the skin can lead to further complications, including scars, chronic post-inflammatory skin changes, and skin atrophy from prolonged use of topical corticosteroids [18].

Mechanism of phototherapy

The fundamental treatment approach for AD involves a multifaceted treatment approach prioritizing non-pharmacological strategies such as avoiding known irritants and using ceramide-containing emollients to enhance skin hydration and barrier functionality. For milder manifestations, topical treatments like glucocorticoids and immunomodulators are effective. However, more severe or persistent cases may require systemic treatments, including short-term glucocorticoids or cyclosporine, alongside continued topical care and skin hydration. Phototherapy emerges as an alternative treatment option, offering a favorable balance of efficacy and safety despite the sparse data on its long-term adverse effects, thereby maintaining its position as a significant therapeutic modality. Phototherapy, which utilizes specific wavelengths of light to treat skin disorders, can be categorized into UV therapy and visible light therapy. UV therapy, leveraging UV light, addresses conditions like psoriasis and AD, whereas visible light therapy uses certain wavelengths within the visible spectrum to treat ailments such as acne and seasonal affective disorder (SAD). UV phototherapy, in particular, provides a localized treatment that circumvents the systemic side effects associated with more profound immunosuppression, cementing its role in dermatological treatment, especially with the advent of biological agents [19-21].

UV therapy's mechanism involves both immediate and delayed effects. The immediate impact includes cellular membrane and DNA damage, activation of transcription factors, and chromophore isomerization, leading to growth arrest and apoptosis. Specifically, UVB radiation directly damages DNA, disrupting transcription and cell division processes in a response known as the phototype I reaction. These alterations can halt cell cycle progression in fibroblasts and epidermal cells [22,23].

UV radiation can cause cellular damage by directly altering the molecular structure of DNA in response to UVB radiation. This alteration inhibits DNA transcription machinery, disrupts the cell cycle, and results in growth arrest in human fibroblasts and epidermal cells, a process known as the phototype I reaction. In essence, the direct absorption of photons by DNA can lead to structural changes in the DNA molecule, ultimately disrupting cell division in response to UV radiation [19,21,23].

The delayed effects of UV therapy predominantly suppress the immune system by impairing both adaptive and innate immune functions. This includes reducing the skin's T-cell population and Langerhans cell count, enhancing T-regulatory cell activity to temper immune responses, and causing oxidative stress that impedes dendritic cell function in T-cell stimulation. This immune modulation results in the decreased production of pro-inflammatory cytokines while increasing IL-10 levels, which has an immunosuppressive role. Furthermore, UV therapy induces apoptosis through mechanisms differing by UVA1 and UVB exposure, affecting cellular membranes and DNA integrity [19,21,23].

Beyond its immunomodulatory effects, UV exposure also influences the skin's structural integrity and function. It promotes the thickening of the stratum corneum, enhancing barrier protection and reducing eczema flare-ups [19].

There's an upregulation of critical barrier proteins, such as filaggrin and involucrin, essential for the skin's protective capabilities and moisture retention [3,22]. Additionally, UV therapy possesses antibacterial properties, especially against *Staphylococcus aureus*, helping decrease bacterial skin colonization and toxin production, thereby improving skin health [24]. Another significant aspect of UV therapy is its role in vitamin D synthesis. UVB rays convert 7-dehydrocholesterol in the skin to pre-vitamin D, which is essential for maintaining immune balance and skin barrier function. AD patients often exhibit vitamin D deficiency, implicating a potential role in the disease's pathophysiology and barrier dysfunction [25].

When planning phototherapy, several considerations are paramount, including disease severity, patient health, treatment cost and accessibility, lesion location and skin type, cancer history, photosensitivity, and the Fitzpatrick skin type. The treatment regimen can be adjusted for chronic or refractory conditions to a regular or maintenance schedule, ensuring therapeutic efficacy over time [26].

Despite its effectiveness in treating AD, phototherapy does have limitations. The necessity for bi-weekly sessions may pose logistical issues for those far from treatment centers. Moreover, it may not be suitable for all patients, particularly those with light-sensitive disorders. The risk levels associated with different phototherapy types also necessitate careful consideration in treatment planning [27].

Adverse effects of phototherapy can range from actinic damage and erythema to more severe outcomes like non-melanoma skin cancer, melanoma, and cataracts, especially with PUVA (psoralen and UVA) therapy. Other potential side effects include photosensitive eruptions, folliculitis, photo-onycholysis, HSV reactivation, and facial hypertrichosis. UVA therapy risks extend to cataract formation, while oral psoralen, used in conjunction with UVA, may cause systemic side effects like nausea and increased photosensitivity [27].

Indications of phototherapy for patients with AD

Phototherapy offers a pivotal treatment for AD, especially when traditional treatments fail. Utilizing narrowband UVB light, it exerts immunomodulatory effects to lessen inflammation and itching, crucial for AD management. It also enhances skin barrier function by increasing essential proteins and lipids. This method not only alleviates pruritus, improving patient well-being by interrupting the itch-scratch cycle, but also requires customization based on individual factors like skin type and disease severity. Administered under medical supervision, the therapy's dosing and frequency are tailored to each patient's unique profile to ensure efficacy and safety [1,3,19,28,29].

Therefore, the application of phototherapy in treating patients with AD is recommended as follows: phototherapy is used as an alternative strategy when primary treatments such as emollients, topical steroids, and topical calcineurin inhibitors prove ineffective and serves as an ongoing treatment approach for individuals dealing with chronic skin conditions; implementation of any form of phototherapy necessitates the direction and continuous monitoring by a physician experienced in phototherapy methods; factors like availability, cost, the patient's skin type, history of skin cancer, and the use of photosensitizing drugs should guide the choice of light therapy; the intensity and schedule of light therapy ought to be scheduled according to the patient's minimal erythema dose (MED) and/or their Fitzpatrick skin type; and in cases where patients are unable to access phototherapy in a medical facility, home phototherapy may be considered, but strictly under the supervision of a qualified physician.

For the effective use of phototherapy in treating AD, the determination of optimal dosing strategies, including MED, is crucial. MED, defined as the smallest amount of light radiation needed to produce visible skin reddening, is foundational for setting initial therapy levels. These doses are adjusted based on individual responses and skin types to balance therapeutic effectiveness against the risk of side effects. Safety protocols are essential to prevent burns and other adverse reactions, with treatment customization according to patient needs and outcomes. The accuracy in applying MED is key to maximizing phototherapy benefits, improving patient outcomes, and ensuring safety [7].

Types of phototherapy used for AD

Phototherapy, also known as photochemotherapy when combined with psoralens, is a valuable treatment modality for AD. Different types of phototherapy use specific wavelengths of light to treat skin conditions, including AD. These treatments can help manage the symptoms of AD and reduce disease activity. Below are some of the key modalities of phototherapy used in AD treatment.

Narrowband UVB (NB-UVB; 311-313 nm) and Broadband UVB (BB-UVB, 290-320 nm)

NB-UVB is considered the preferred choice for phototherapy in AD due to its efficacy, good tolerability, and minimal risk. NB-UVB has been widely selected as the best therapeutic option because it effectively suppresses the Th2, Th22, and Th1 pathways, reduces T cells and cytokines, and normalizes cell differentiation, epidermal hyperplasia, and the expression of barrier proteins. Dosing guidelines for NB-UVB are illustrated in Table 2, Table 3, Table 4, and Table 5 [28].

**Table 2:** Dosing guidelines for NV-UVB according to skin type NV-UVB: narrowband ultraviolet B

Skin type	Initial UVB dose (mJ/cm²)	UVB increase after each treatment (mJ/cm²)	Maximum dose (mJ/cm²)
I	130	15	2000
II	220	25	2000
III	260	40	3000
IV	330	45	3000
V	350	60	5000
VI	400	65	5000

**Table 3:** Dosing guidelines for NB-UVB according to MED MED: minimal erythema dose; NV-UVB: narrowband ultraviolet B

Stage	Dosing protocol
Initial UVB	50% of MED
Treatments 1-20	Increase by 10% of the initial MED
Treatment ≥21	Increase as ordered by a physician

**Table 4:** Protocol for missed treatments

Missed treatment duration	Protocol
4-7 days	Keep dose same
1-2 weeks	Decrease dose by 25%
2-3 weeks	Decrease dose by 50% or start over
3-4 weeks	Start over

**Table 5:** Maintenance therapy for NB-UVB after >95% clearance Administered 3-5×/week The variability in the MED for NB-UVB among different skin types necessitates routine MED testing. Regular calibration of the UVB machine, ideally weekly, is essential due to the gradual decrease in the efficacy of UVB lamps over time. Without consistent measurement and adjustment of the UV output, there's a possibility that clinicians might overestimate the dose being delivered, leading to administering lower doses than intended. The number of phototherapy sessions required weekly for effective ongoing treatment, as well as the length of this maintenance phase, differs widely from one individual to another. While an ideal treatment plan would allow for a gradual decrease in the frequency of phototherapy, many patients might require continuous NB-UVB phototherapy, usually once a week, to maintain long-term effectiveness. MED: minimal erythema dose; NV-UVB: narrowband ultraviolet B

Frequency	Duration	Protocol
1×/week	4 weeks	NB-UVB, keep dose same
1×/every 2 weeks	4 weeks	NB-UVB, decrease dose by 25%
1×/every 4 weeks	Indefinite	NB-UVB, 50% of the highest dose

BB-UVB, on the other hand, is less commonly suggested because it is more likely to cause erythematous (redness of the skin) effects and is less effective than NB-UVB [1,3,7,19,29]. Dosing guidelines for BB-UVB are illustrated in Table 6, Table 7, and Table 8 [28].

**Table 6:** BB-UVB dosing according to skin type BB-UVB: broadband ultraviolet B

Skin type	Initial UVB dose (mJ/cm²)	UVB increase after each treatment (mJ/cm²)
I	20	5
II	25	10
III	30	15
IV	40	20
V	50	25
VI	60	30

**Table 7:** Broadband UVB dosing adjustments according to MED MED: minimal erythema dose; BB-UVB: broadband ultraviolet B

Treatment stage	Dosing adjustment
Initial UVB	50% of MED
Treatments 1-10	Increase by 25% of the initial MED
Treatments 11-20	Increase by 10% of the initial MED
Treatment ≥21	Increase as ordered by a physician

**Table 8:** Protocol for missed BB-UVBB treatments Administered 3-5×/week BB-UVB: broadband ultraviolet B

Missed treatment duration	Protocol
4-7 days	Keep dose same
1-2 weeks	Decrease dose by 50%
2-3 weeks	Decrease dose by 75%
3-4 weeks	Start over

Combined UVA/UVB (280-400 nm)

This modality involves the use of a single device that emits both UVA and UVB radiation. It can be administered as simultaneous or subsequent emissions. UVAB phototherapy has become less common in recent years due to limited effectiveness and accessibility [1,3,6,7,19,29].

UVA and UVA1 (340-400 nm)

UVA therapy, including UVA1, is widely available and less expensive compared to other phototherapy modalities. UVA1, in particular, has shown effectiveness in reducing clinical symptoms of AD, especially in chronic cases and acute flare-ups. UVA1 can be administered at different doses, including high doses, medium doses, and low doses, depending on the severity of AD [1,3,6,7,19,29].

PUVA

PUVA is a second-choice phototherapy treatment modality for AD. It involves the use of psoralens (8-methoxy psoralen) in combination with synthetic long-wave UVA radiation. Psoralens can be administered orally (systemic PUVA), topically (cream-PUVA), or through water delivery (balneotherapy).

PUVA works by causing DNA damage, nucleotide depletion in peripheral blood leukocytes, and the activation of polyADP-ribosylation, ultimately leading to T-cell death. Recent studies have suggested that PUVA treatment may help reduce itching and clinical severity scores in AD patients by regulating epidermal nerve density and the expression of specific axonal molecules like semaphorin 3A (Sema3A) and nerve growth factor (NGF). However, long-term use of PUVA may have carcinogenic effects and should be used with caution [1,3,6,19,29]. Dosing of UVA radiation for oral PUVA is illustrated in Table 9 [28].

**Table 9:** Dosing of UVA radiation for oral PUVA PUVA: psoralen and ultraviolet A

Skin types	Initial dose (J/cm^2^)	Increments (J/cm^2^)	Maximum dose (J/cm^2^)
Ⅰ	0.5	0.5	8
Ⅱ	1.0	0.5	8
Ⅲ	1.5	1.0	12
Ⅳ	2.0	1.0	12
Ⅴ	2.5	1.5	20
Ⅵ	3.0	1.5	20

Overall, phototherapy offers several options for managing AD, and the choice of modality depends on the patient's condition, treatment goals, and potential side effects. It is essential for healthcare providers to consider these factors when recommending phototherapy for AD patients.

Other modalities of treatment for AD

In the comprehensive management of AD, it is critical to distinguish between the roles of topical treatments and systemic/biological therapies. This distinction not only elucidates the diverse therapeutic arsenal available to clinicians but also underscores the tailored approach necessary for individual patient scenarios. The following discussion will delve into the indications, efficacy, and safety profiles of each treatment type, highlighting their unique contributions to AD management and patient care.

Topical Treatment

Topical corticosteroids (TCS): TCS represent the first-line anti-inflammatory therapy for managing AD in both adults and children. These medications exert their effects on various immune cells, including T lymphocytes, monocytes, macrophages, and dendritic cells. TCS impair antigen processing and inhibit the release of cytokines that promote inflammation. They are typically introduced into the treatment regimen when regular moisturizing and proper skincare practices fail to improve lesions. TCS usage is associated with local adverse effects, but the intermittent and correct administration of these medications carries little risk. Comprehensive analyses have revealed an overall favorable safety profile for TCS [1,3,26,30].

Topical calcineurin inhibitors (TCI): Another class of anti-inflammatory drugs used in AD management is TCI. These naturally occurring agents, derived from the bacterium *Streptomyces*, act by preventing calcineurin-dependent T-cell activation. This inhibition leads to the reduced production of pro-inflammatory cytokines and mediators involved in the AD inflammatory response. TCI have also been shown to impact mast cell activation, and tacrolimus, a TCI, reduces both the quantity and costimulatory capacity of epidermal dendritic cells. Unlike topical steroids, TCI can be safely applied to areas with thinner skin, such as the face (including the eyelids), neck, and groin, without causing skin atrophy. Common side effects include localized reactions like burning, stinging, and itching [1,3,17].

Topical phosphodiesterase 4 (PDE4) inhibitors: PDE4 inhibitors have emerged as a potential treatment option for AD. These nonsteroidal anti-inflammatory drugs (NSAIDs) prevent skin atrophy and the breakdown of the epithelial barrier that can occur with corticosteroid therapy. They also modulate the inflammatory response by targeting PDE4, a crucial regulator of inflammatory cytokine production in AD. PDE4 inhibitors reduce the expression of pro-inflammatory cytokines by increasing cyclic adenosine monophosphate levels in AD patients [1,3,31,31].

Systemic Agent

Methotrexate (MTX): Systemic treatments like MTX are employed to manage AD. MTX exerts an anti-inflammatory effect by elevating intracellular and extracellular adenosine levels, a purine nucleoside with anti-inflammatory properties. It accomplishes this by inhibiting 5-aminoimidazole-4-carboxamide ribonucleotide transformylase, reducing inflammation by neutralizing free radicals, and decreasing the production of malondialdehyde-acetaldehyde protein adducts. However, MTX use may lead to hepatotoxicity, hematologic toxicity, gastrointestinal issues, and, in cases of poor renal function, excessive MTX levels [1,3,32].

Cyclosporine: Cyclosporine A is another systemic treatment option. It suppresses the infiltration of immunological cells like CD4+ T cells, mast cells, and eosinophils. Additionally, it reduces the growth of intraepidermal nerve fibers, diminishing the sensation of itch stimuli. However, its use may be limited in individuals with liver or renal disease who are taking colchicine, as it is contraindicated [3,33].

Janus kinase (JAK) inhibitors: In the realm of biological treatments, JAK inhibitors have emerged as a novel approach to AD treatment, owing to their low molecular weight that allows for both topical and systemic administration. Examples of JAK inhibitors include delgocitinib, ruxolitinib, and tofacitinib in topical formulations and baricitinib, upadacitinib, abrocitinib, and gusacitinib in systemic formulations. Upadacitinib and abrocitinib, oral selective JAK-1 inhibitors, have demonstrated significant clinical benefits in moderate to severe AD patients and have been well tolerated [34].

Biologics

Dupilumab: Dupilumab, a monoclonal antibody, represents a significant advancement in the treatment of moderate to severe AD for patients who do not respond adequately to topical treatments or when such treatments are unsuitable. Dupilumab binds to the IL-4Rα chain, present in both the IL-4 and IL-13 receptors. This action blocks both the IL-4 and IL-13 pathways, inhibiting the central Th2 response crucial in AD pathogenesis. Dupilumab also effectively inhibits Th2-associated chemokines, reduces mRNA expression of hyperplasia-related genes, and inhibits IL-17/IL-22-modulated genes [35].

Upadacitinib: Upadacitinib, a selective JAK-1 inhibitor, has shown promise in treating moderate to severe atopic dermatitis. In a 16-week trial, it demonstrated a dose-response efficacy, with the highest dose offering the greatest clinical benefit without observed dose-limiting toxicity. This positions upadacitinib as a potentially effective oral monotherapy for patients inadequately controlled by topical treatments, underscoring its role in the evolving landscape of atopic dermatitis management [36]

## Conclusions

Our review highlights phototherapy, including NB-UVB and UVA1, as a key treatment for AD, due to its safety and effectiveness, especially in treatment-resistant cases. It advocates for phototherapy's integration into AD management strategies to enhance outcomes. The review also emphasizes the need for clinicians to customize phototherapy approaches and calls for further research on its long-term effects, mechanisms, and cost-effectiveness in diverse populations. This research could lead to optimized treatments and new therapeutic directions, underscoring the ongoing importance of phototherapy in providing tailored, effective care for AD patients.

## References

[1] Bolognia J, Jorizzo JL, Rapini RP (2008). Dermatology. https://search.worldcat.org/title/dermatology/oclc/212399895.

[2] James WD, Elston DM, Berger TG, Andrews GC (2011). Andrews' diseases of the skin : clinical dermatology. https://search.worldcat.org/title/663444979.

[3] Wolff K, Fitzpatrick TB, Goldsmith LA (2008). Fitzpatrick's dermatology in general medicine. https://search.worldcat.org/title/192043562.

[4] Kim JE, Kim HJ, Lew BL (2015). Consensus guidelines for the treatment of atopic dermatitis in Korea (part I): general management and topical treatment. Ann Dermatol.

[5] Davis DM, Borok J, Udkoff J, Lio P, Spergel J (2017). Atopic dermatitis: phototherapy and systemic therapy. Semin Cutan Med Surg.

[6] Vangipuram R, Feldman SR (2016). Ultraviolet phototherapy for cutaneous diseases: a concise review. Oral Dis.

[7] Patrizi A, Raone B, Ravaioli GM (2015). Management of atopic dermatitis: safety and efficacy of phototherapy. Clin Cosmet Investig Dermatol.

[8] Maspero J, De Paula Motta Rubini N, Zhang J (2023). Epidemiology of adult patients with atopic dermatitis in AWARE 1: a second international survey. World Allergy Organ J.

[9] Silverberg JI, Barbarot S, Gadkari A (2021). Atopic dermatitis in the pediatric population: a cross-sectional, international epidemiologic study. Ann Allergy Asthma Immunol.

[10] Elezbawy B, Fasseeh AN, Fouly E (2022). The humanistic and economic burden of atopic dermatitis among adults and adolescents in Saudi Arabia. J Med Econ.

[11] Guttman-Yassky E, Waldman A, Ahluwalia J, Ong PY, Eichenfield LF (2017). Atopic dermatitis: pathogenesis. Semin Cutan Med Surg.

[12] Koga C, Kabashima K, Shiraishi N, Kobayashi M, Tokura Y (2008). Possible pathogenic role of Th17 cells for atopic dermatitis. J Invest Dermatol.

[13] Osawa R, Akiyama M, Shimizu H (2011). Filaggrin gene defects and the risk of developing allergic disorders. Allergol Int.

[14] Oetjen LK, Mack MR, Feng J (2017). Sensory neurons co-opt classical immune signaling pathways to mediate chronic itch. Cell.

[15] Tada J (2002). Diagnostic standard for atopic dermatitis. JMA Policies.

[16] Eichenfield LF, Tom WL, Chamlin SL (2014). Guidelines of care for the management of atopic dermatitis: section 1. Diagnosis and assessment of atopic dermatitis. J Am Acad Dermatol.

[17] Frazier W, Bhardwaj N (2020). Atopic dermatitis: diagnosis and treatment. Am Fam Physician.

[18] Berke R, Singh A, Guralnick M (2012). Atopic dermatitis: an overview. Am Fam Physician.

[19] Ganzetti G, Campanati A, Offidani A (2017). Phototherapy in dermatology. https://search.worldcat.org/title/989513230.

[20] Gambichler T (2009). Management of atopic dermatitis using photo(chemo)therapy. Arch Dermatol Res.

[21] Vieyra-Garcia PA, Wolf P (2021). A deep dive into UV-based phototherapy: mechanisms of action and emerging molecular targets in inflammation and cancer. Pharmacol Ther.

[22] Borgia F, Li Pomi F, Vaccaro M, Alessandrello C, Papa V, Gangemi S (2022). Oxidative stress and phototherapy in atopic dermatitis: mechanisms, role, and future perspectives. Biomolecules.

[23] Kemény L, Varga E, Novak Z (2019). Advances in phototherapy for psoriasis and atopic dermatitis. Expert Rev Clin Immunol.

[24] Burns EM, Ahmed H, Isedeh PN (2019). Ultraviolet radiation, both UVA and UVB, influences the composition of the skin microbiome. Exp Dermatol.

[25] Juzeniene A, Grigalavicius M, Juraleviciute M, Grant WB (2016). Phototherapy and vitamin D. Clin Dermatol.

[26] Eichenfield LF, Tom WL, Berger TG (2014). Guidelines of care for the management of atopic dermatitis: section 2. Management and treatment of atopic dermatitis with topical therapies. J Am Acad Dermatol.

[27] Valejo Coelho MM, Apetato M (2016). The dark side of the light: phototherapy adverse effects. Clin Dermatol.

[28] Sidbury R, Davis DM, Cohen DE (2014). Guidelines of care for the management of atopic dermatitis: section 3. Management and treatment with phototherapy and systemic agents. J Am Acad Dermatol.

[29] Dogra S, Mahajan R (2015). Phototherapy for atopic dermatitis. Indian J Dermatol Venereol Leprol.

[30] Martín-Santiago A, Puig S, Arumi D, Rebollo Laserna FJ (2022). Safety profile and tolerability of topical phosphodiesterase 4 inhibitors for the treatment of atopic dermatitis: a systematic review and meta-analysis. Curr Ther Res Clin Exp.

[31] Yamamura K, Nakahara T (2022). The dawn of a new era in atopic dermatitis treatment. J Clin Med.

[32] Nedelcu RI, Balaban M, Turcu G (2019). Efficacy of methotrexate as anti-inflammatory and anti-proliferative drug in dermatology: three case reports. Exp Ther Med.

[33] Toyama S, Tominaga M, Takamori K (2022). Treatment options for troublesome itch. Pharmaceuticals (Basel).

[34] Salvati L, Cosmi L, Annunziato F (2021). From emollients to biologicals: targeting atopic dermatitis. Int J Mol Sci.

[35] Deleanu D, Nedelea I (2019). Biological therapies for atopic dermatitis: an update. Exp Ther Med.

[36] Guttman-Yassky E, Thaçi D, Pangan AL (2020). Upadacitinib in adults with moderate to severe atopic dermatitis: 16-week results from a randomized, placebo-controlled trial. J Allergy Clin Immunol.

